# Supplementary figures and images for: HER2-driven mammary tumorigenesis enhances bioenergetics despite reductions in mitochondrial content
Source: eLife. 2026 May 6;14:RP104079. doi: 10.7554/eLife.104079 (PMC13148823; doi:10.7554/eLife.104079)

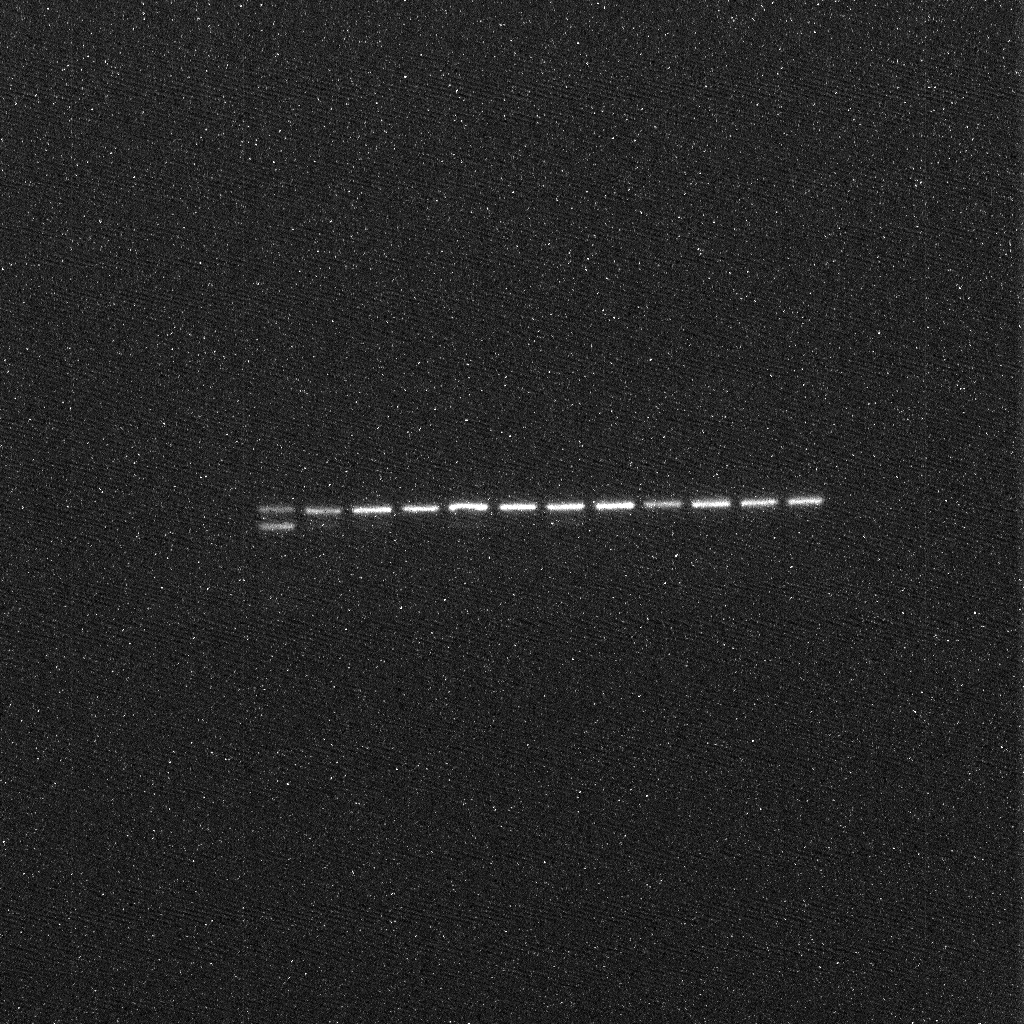

Supplement: Figure 1—source data 2. [file elife-104079-fig1-data2.zip › AKT.tif]

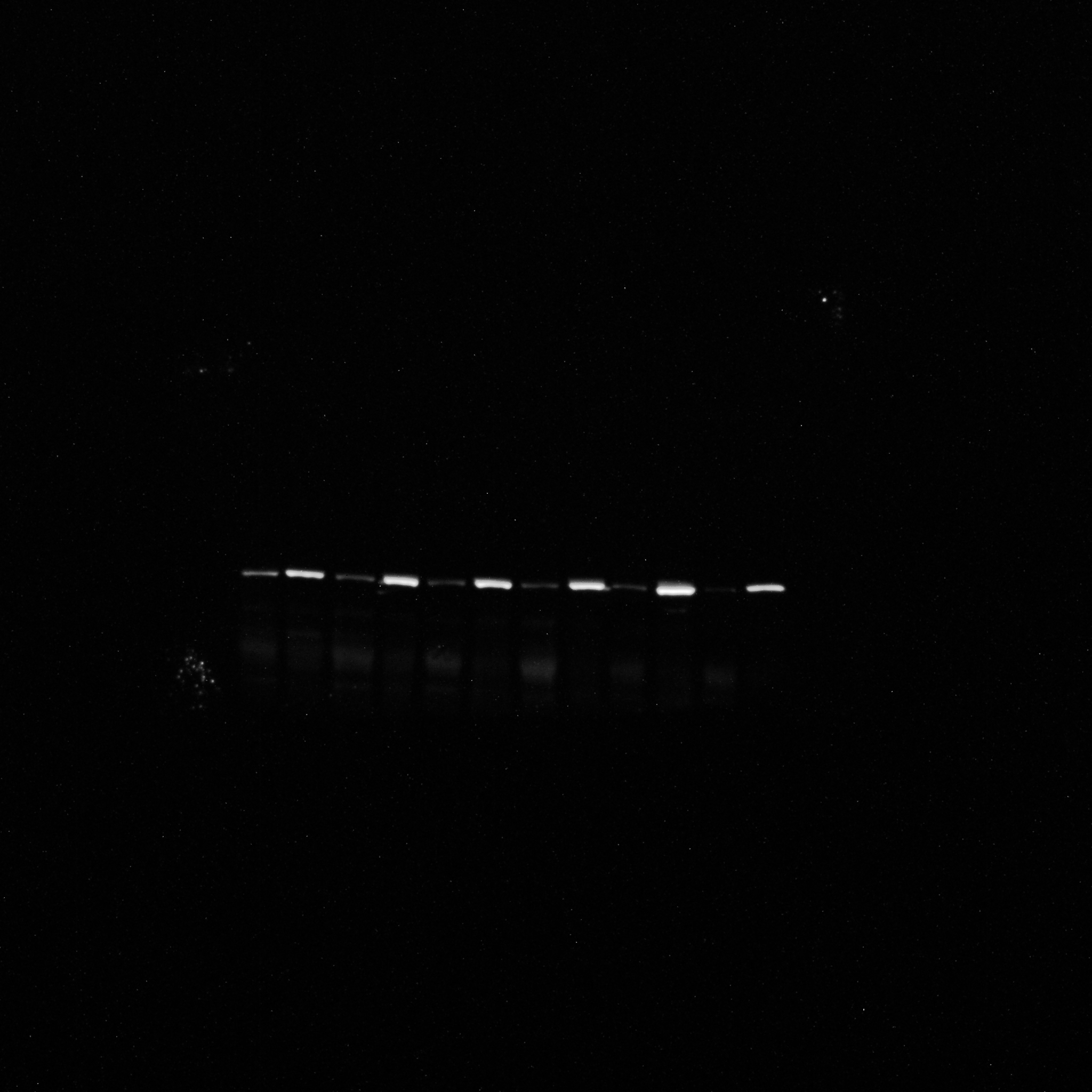

Supplement: Figure 1—source data 2. [file elife-104079-fig1-data2.zip › eEF2.tif]

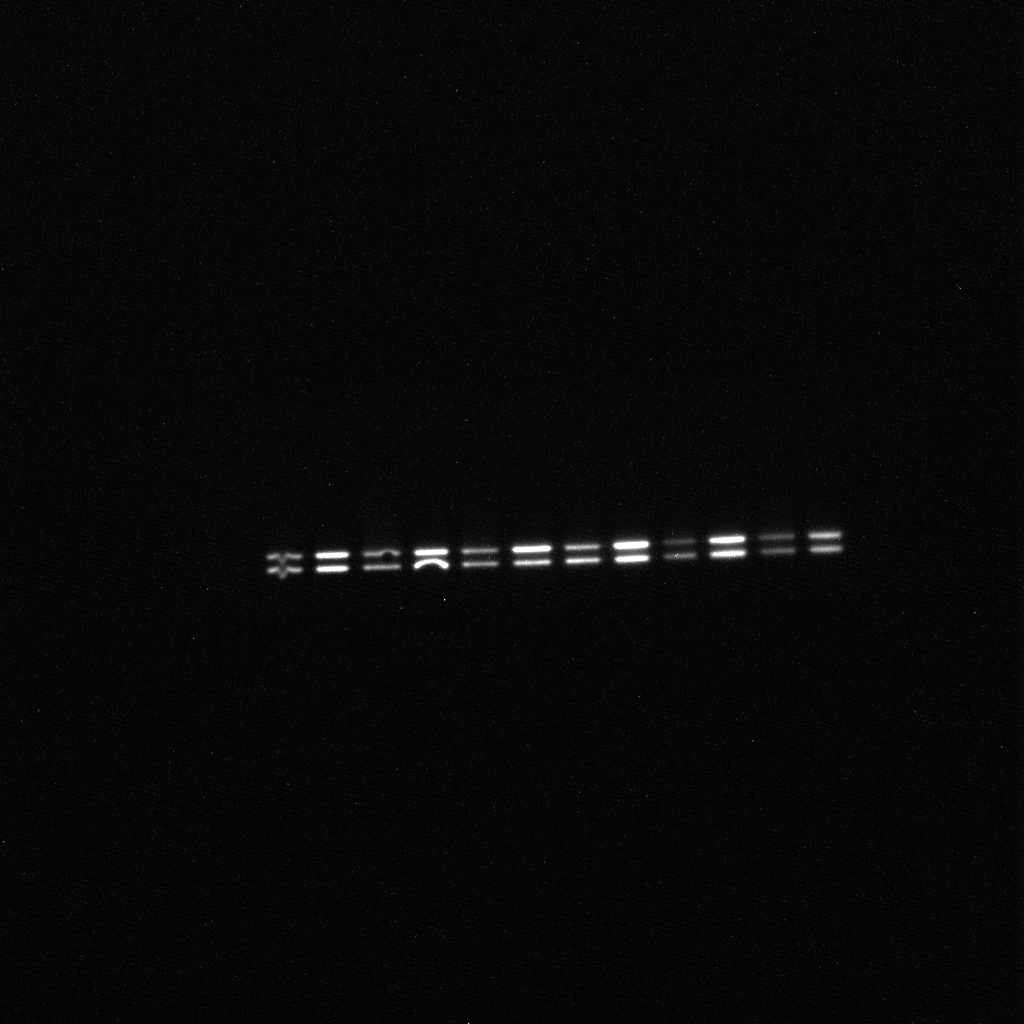

Supplement: Figure 1—source data 2. [file elife-104079-fig1-data2.zip › ERK1_2.tif]

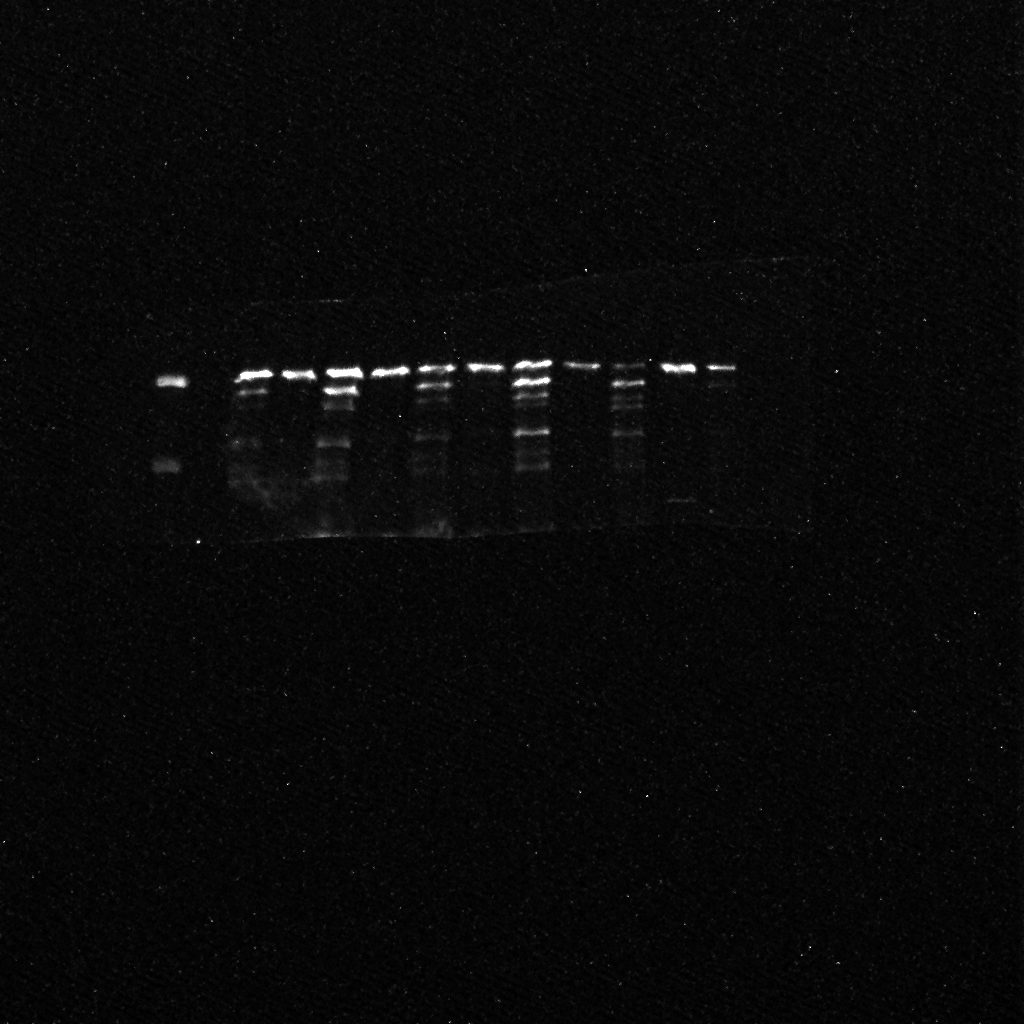

Supplement: Figure 1—source data 2. [file elife-104079-fig1-data2.zip › mTOR.tif]

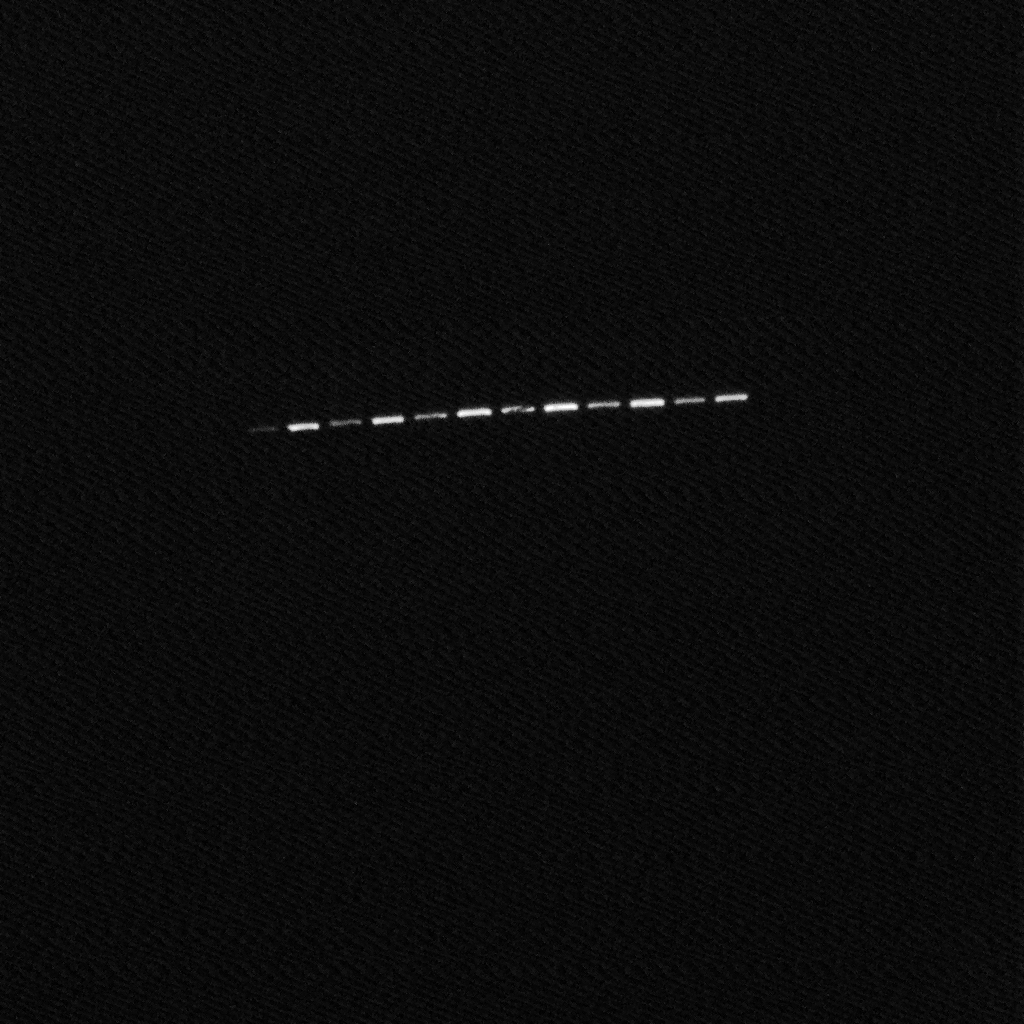

Supplement: Figure 1—source data 2. [file elife-104079-fig1-data2.zip › p38_MAPK.tif]

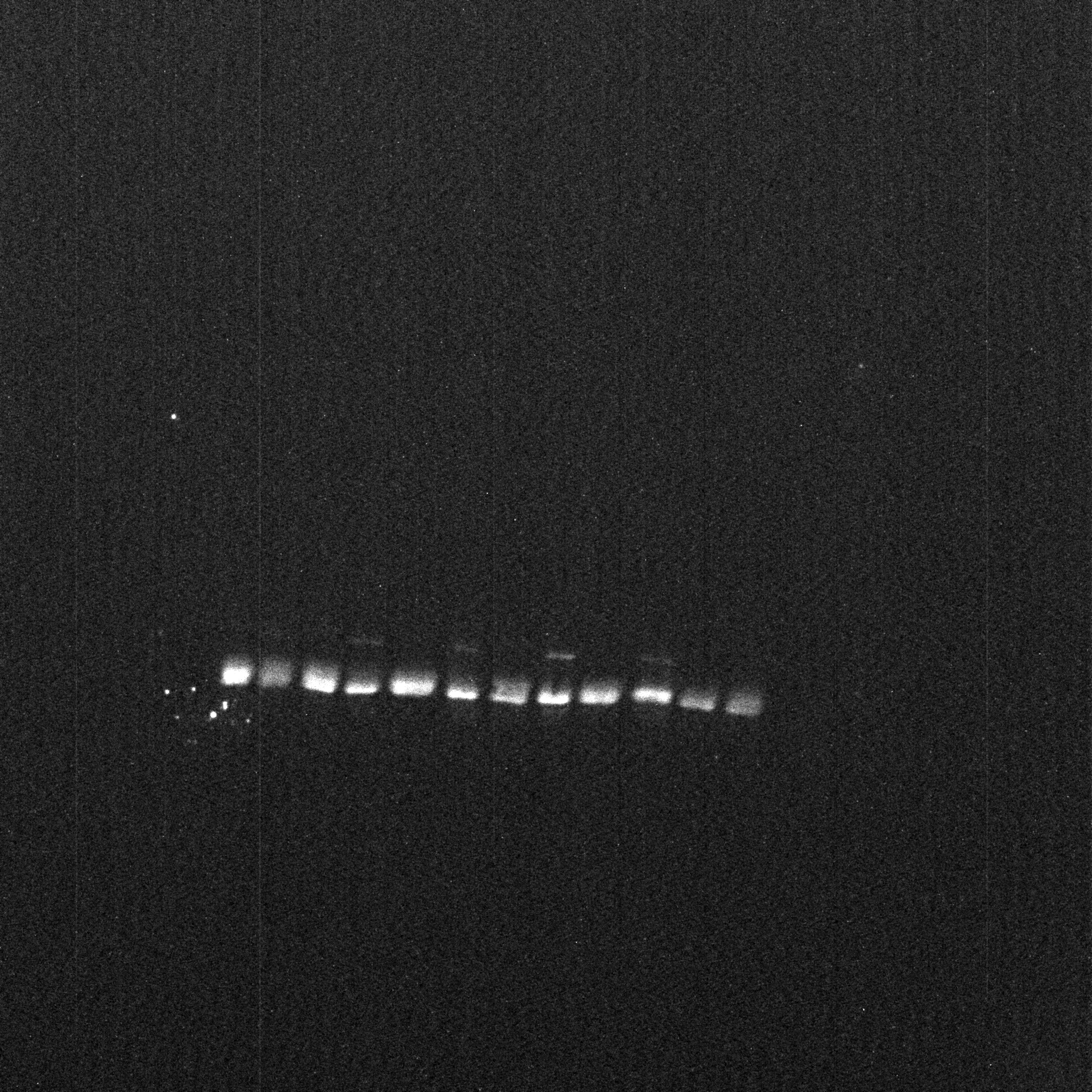

Supplement: Figure 1—source data 2. [file elife-104079-fig1-data2.zip › p70_S6K1.tif]

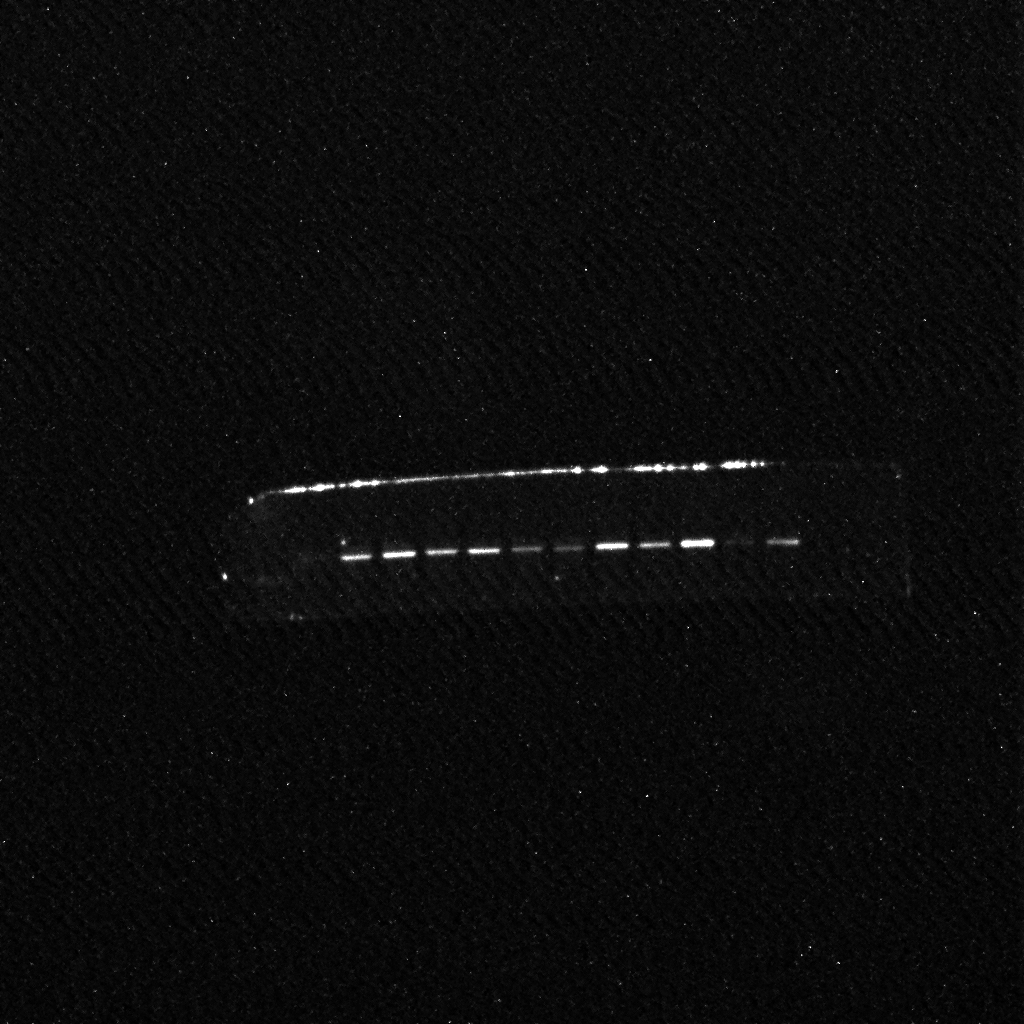

Supplement: Figure 1—source data 2. [file elife-104079-fig1-data2.zip › p-AKT.tif]

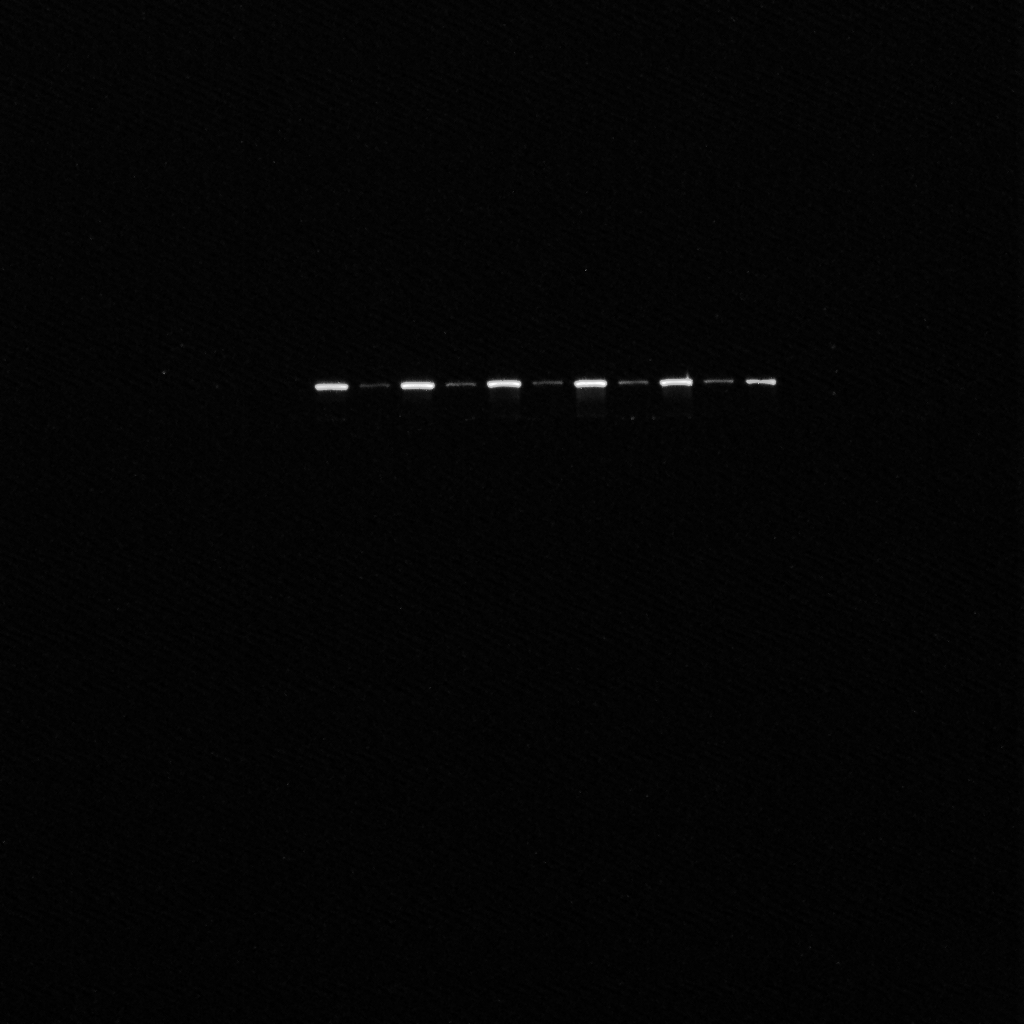

Supplement: Figure 1—source data 2. [file elife-104079-fig1-data2.zip › p-eEF2.tif]

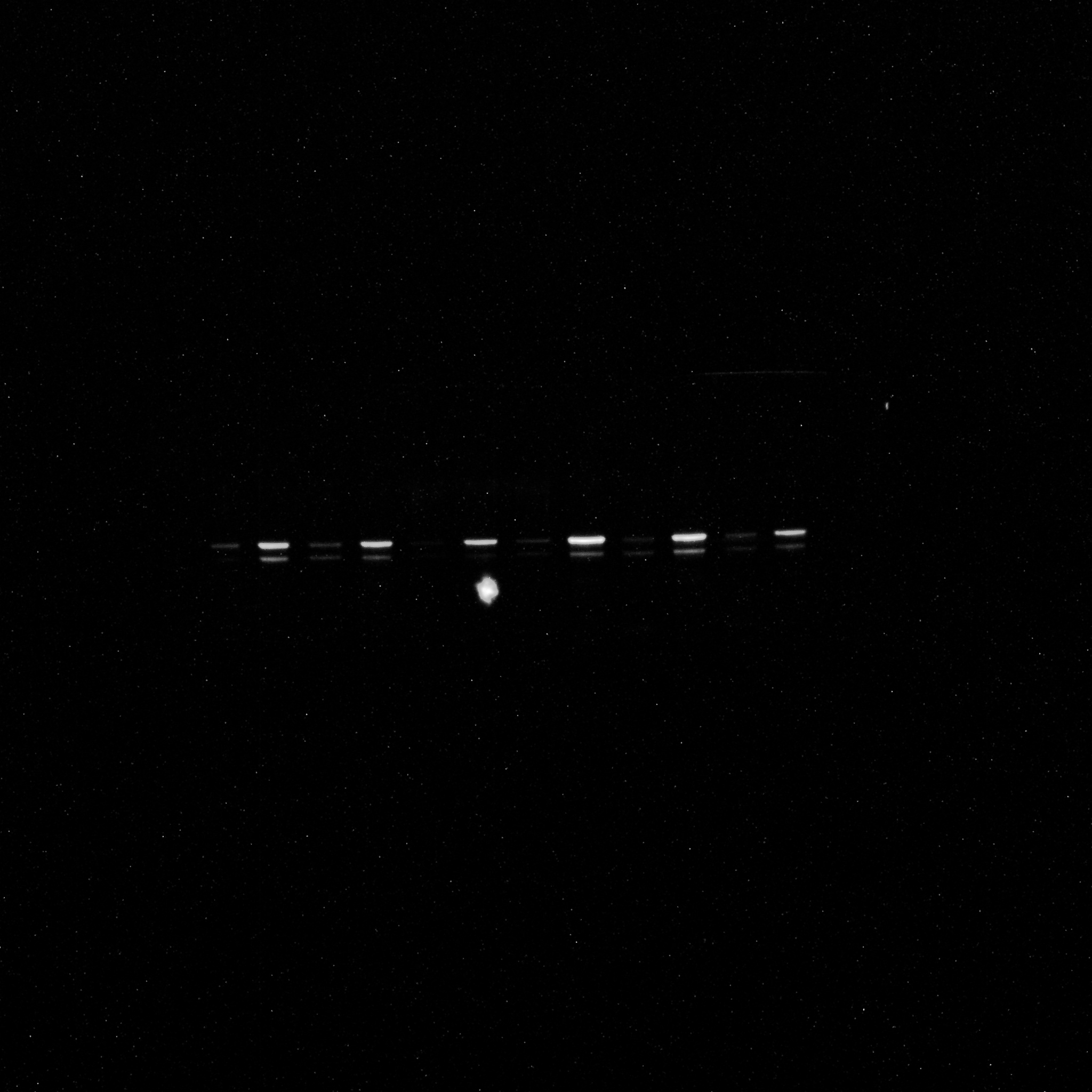

Supplement: Figure 1—source data 2. [file elife-104079-fig1-data2.zip › p-ERK1_2.tif]

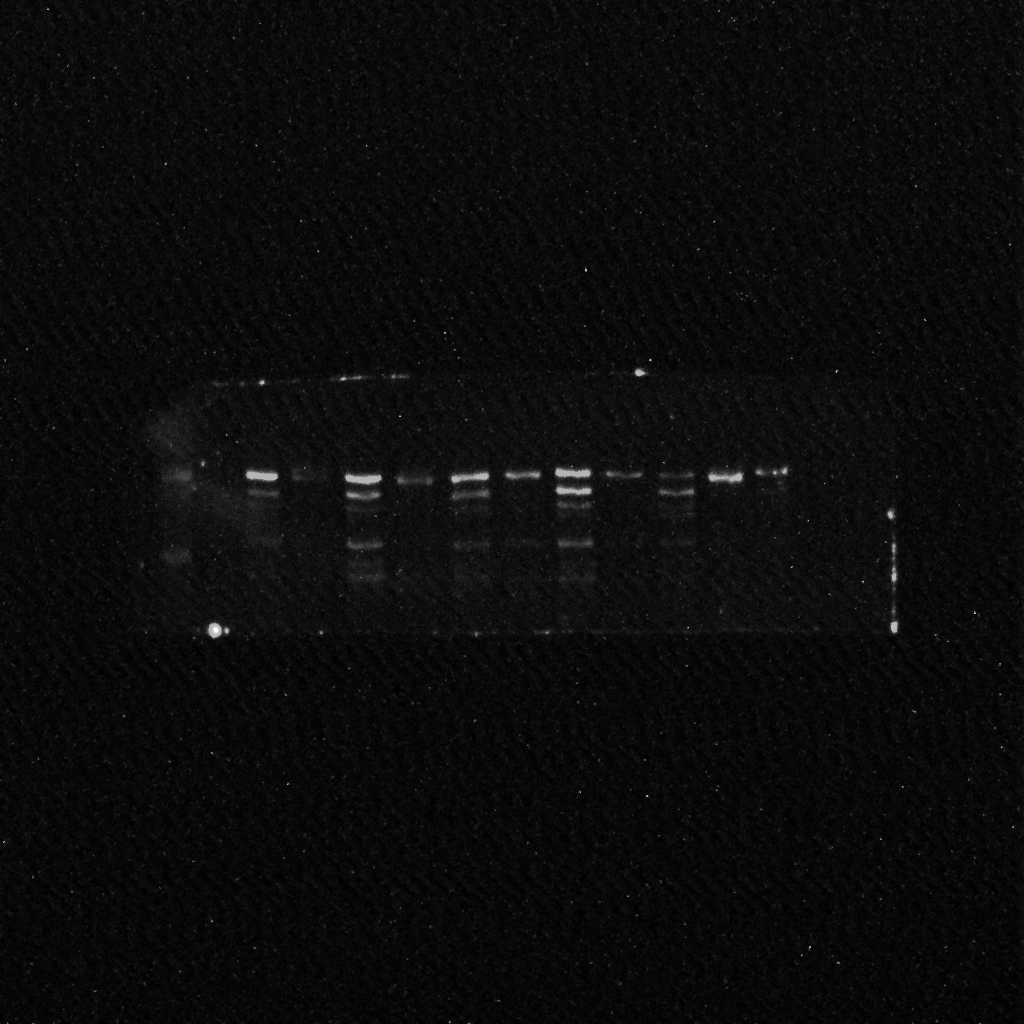

Supplement: Figure 1—source data 2. [file elife-104079-fig1-data2.zip › p-mTOR.tif]

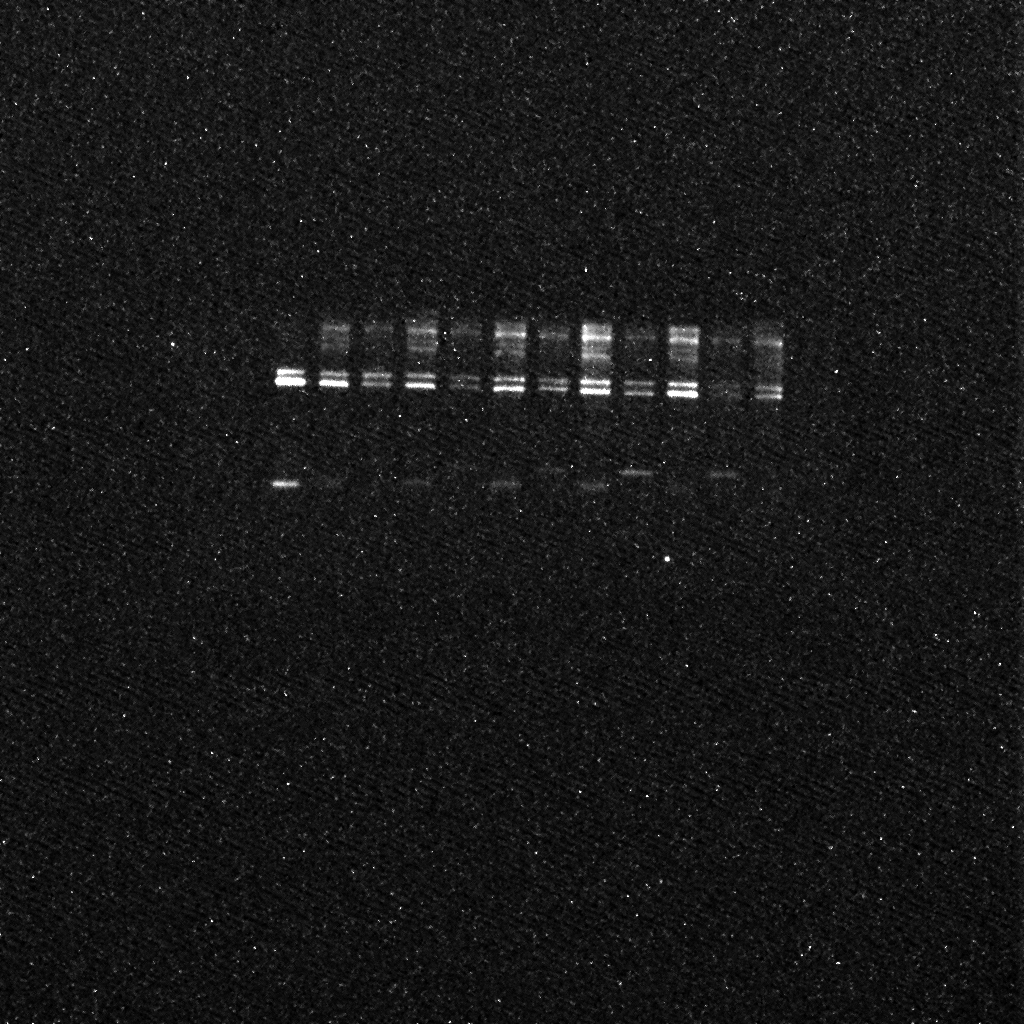

Supplement: Figure 1—source data 2. [file elife-104079-fig1-data2.zip › p-p38_MAPK.tif]

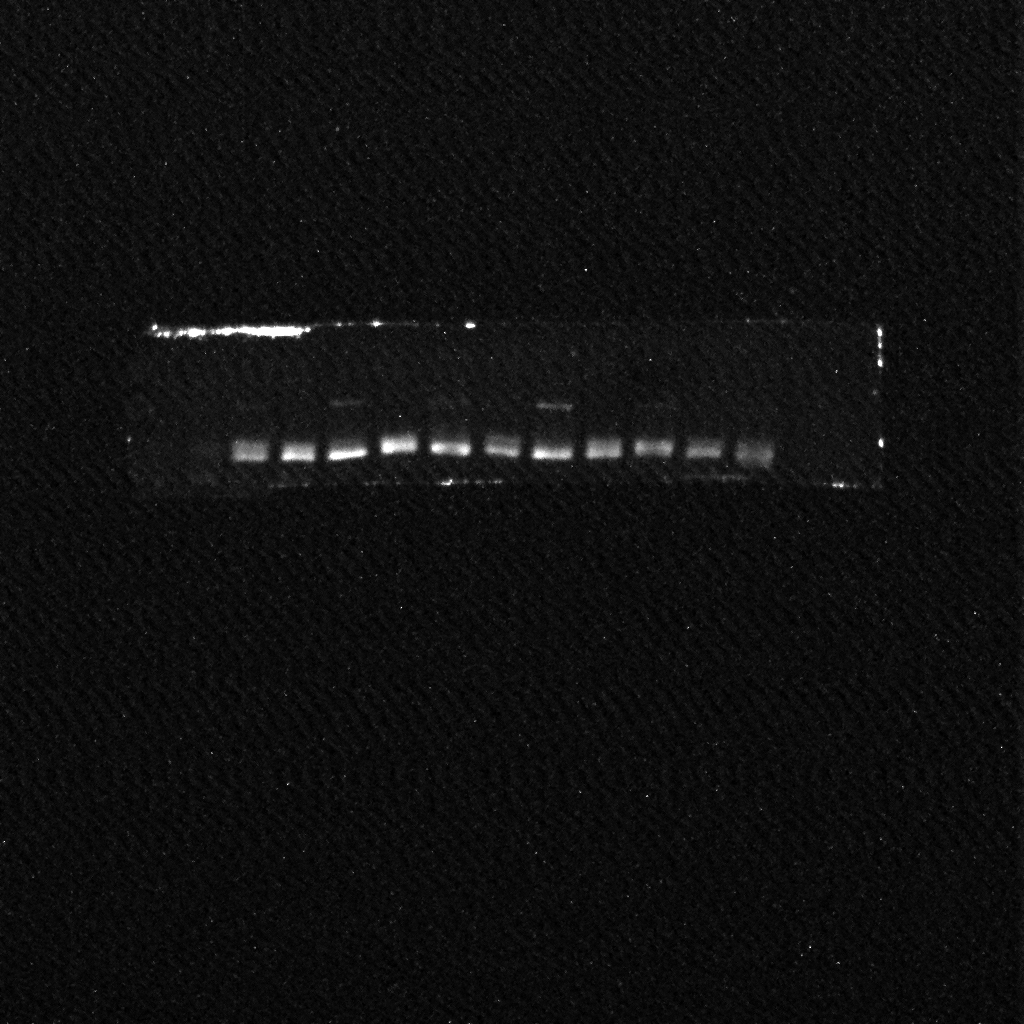

Supplement: Figure 1—source data 2. [file elife-104079-fig1-data2.zip › p-p70_S6K1.tif]

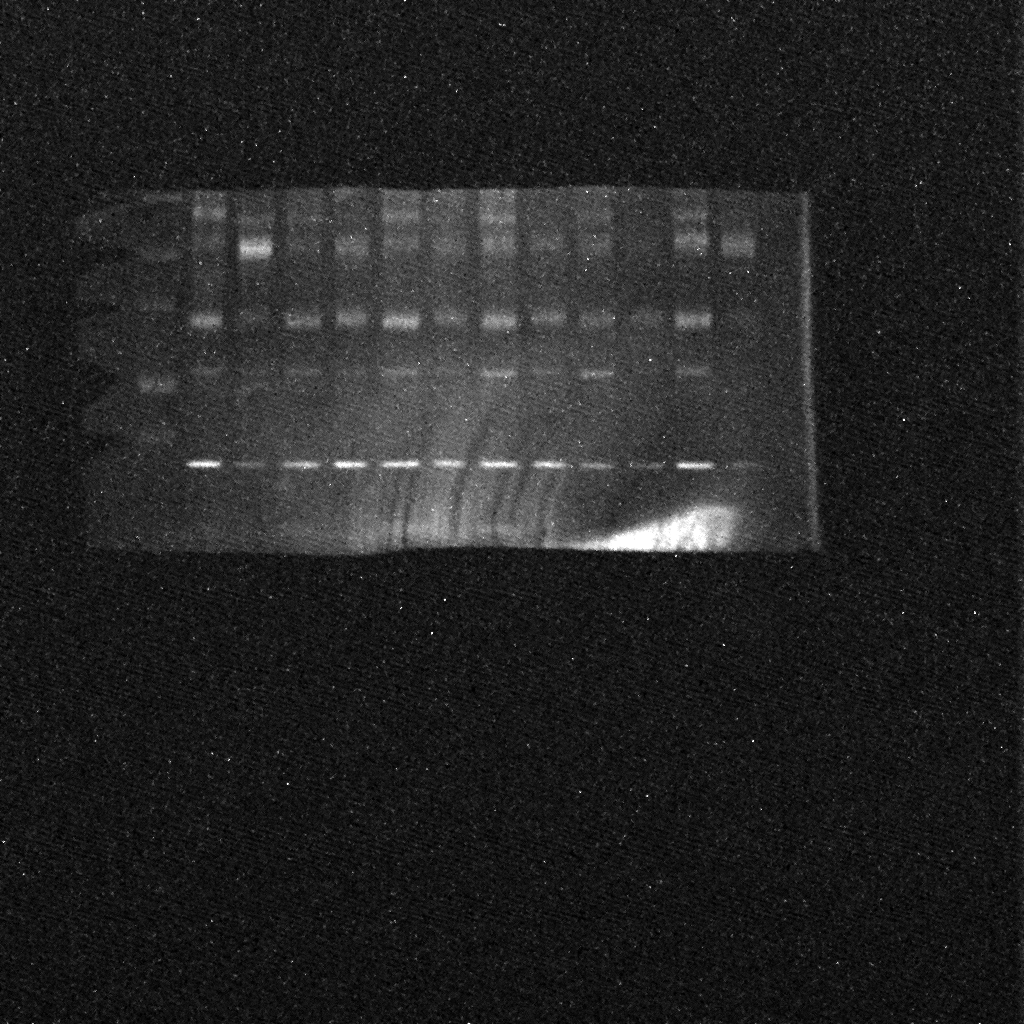

Supplement: Figure 5—figure supplement 2—source data 2. [file elife-104079-fig5-figsupp2-data2.zip › Complex_II_IV.tif]
