# Supplementary material for: HER2-driven mammary tumorigenesis enhances bioenergetics despite reductions in mitochondrial content
Source: eLife. 2026 May 6;14:RP104079. doi: 10.7554/eLife.104079 (PMC13148823; doi:10.7554/eLife.104079)
Supplement: Figure 1—figure supplement 1—source data 1. [file elife-104079-fig1-figsupp1-data1.zip › Figure 1-Figure supplement 1-Source Data 1.pptx]

## Slide 1
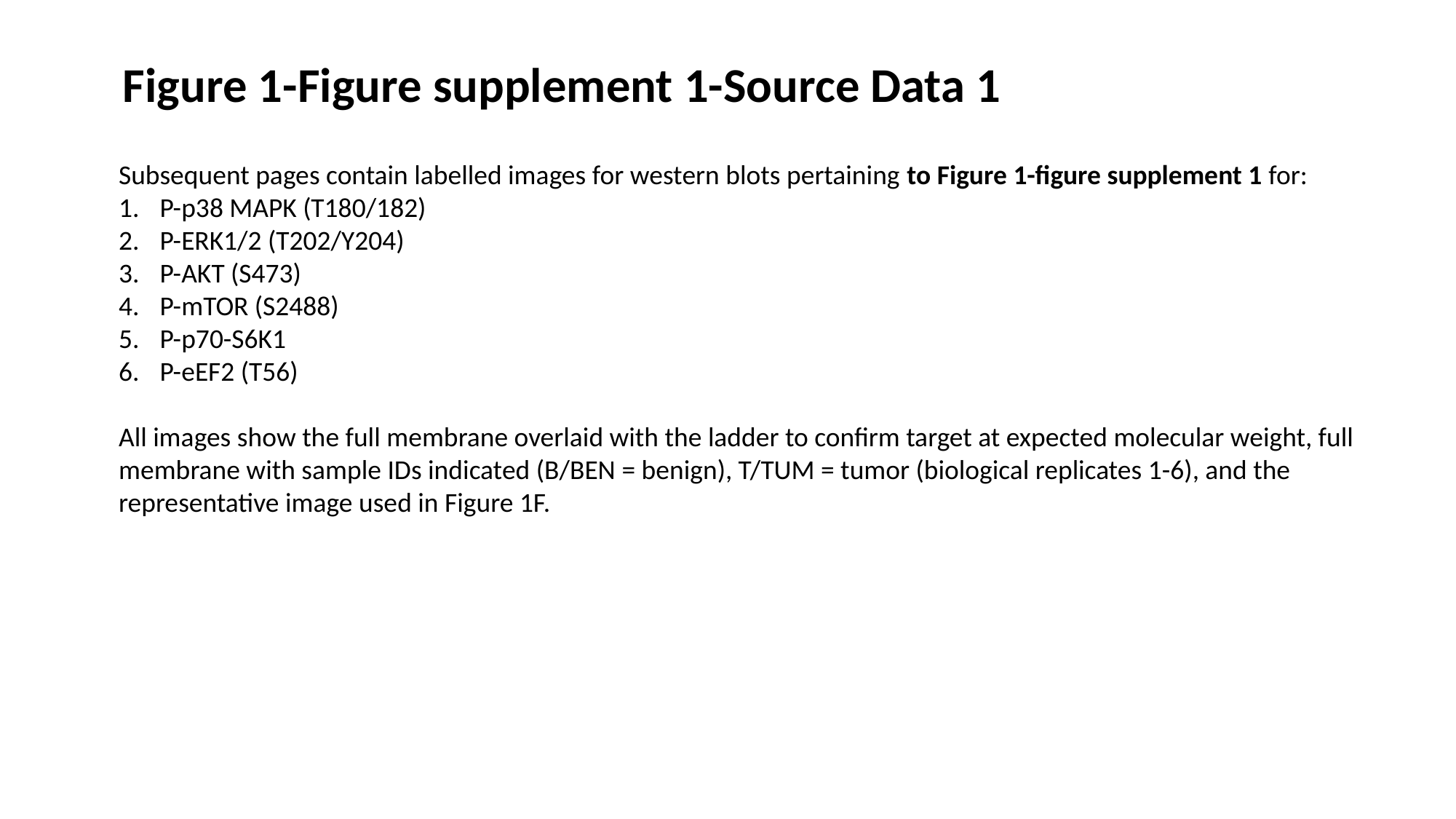

Figure 1-Figure supplement 1-Source Data 1
Subsequent pages contain labelled images for western blots pertaining to Figure 1-figure supplement 1 for:
P-p38 MAPK (T180/182)
P-ERK1/2 (T202/Y204)
P-AKT (S473)
P-mTOR (S2488)
P-p70-S6K1
P-eEF2 (T56)
All images show the full membrane overlaid with the ladder to confirm target at expected molecular weight, full membrane with sample IDs indicated (B/BEN = benign), T/TUM = tumor (biological replicates 1-6), and the representative image used in Figure 1F.

## Slide 2
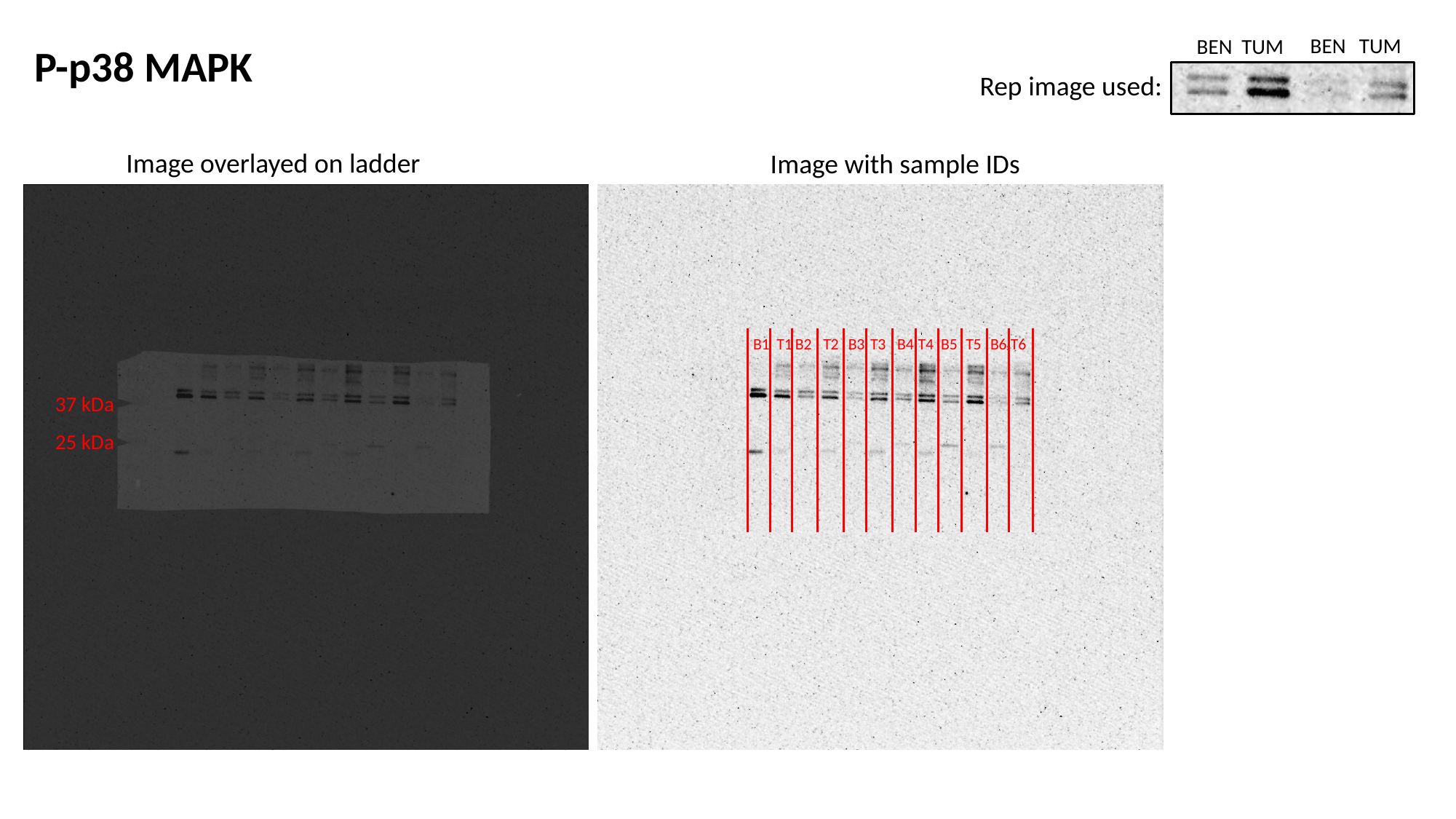

BEN
TUM
TUM
BEN
P-p38 MAPK
Rep image used:
Image overlayed on ladder
Image with sample IDs
B1
T1
B2
T2
B3
T3
B4
T4
B5
T5
B6
T6
37 kDa
25 kDa

## Slide 3
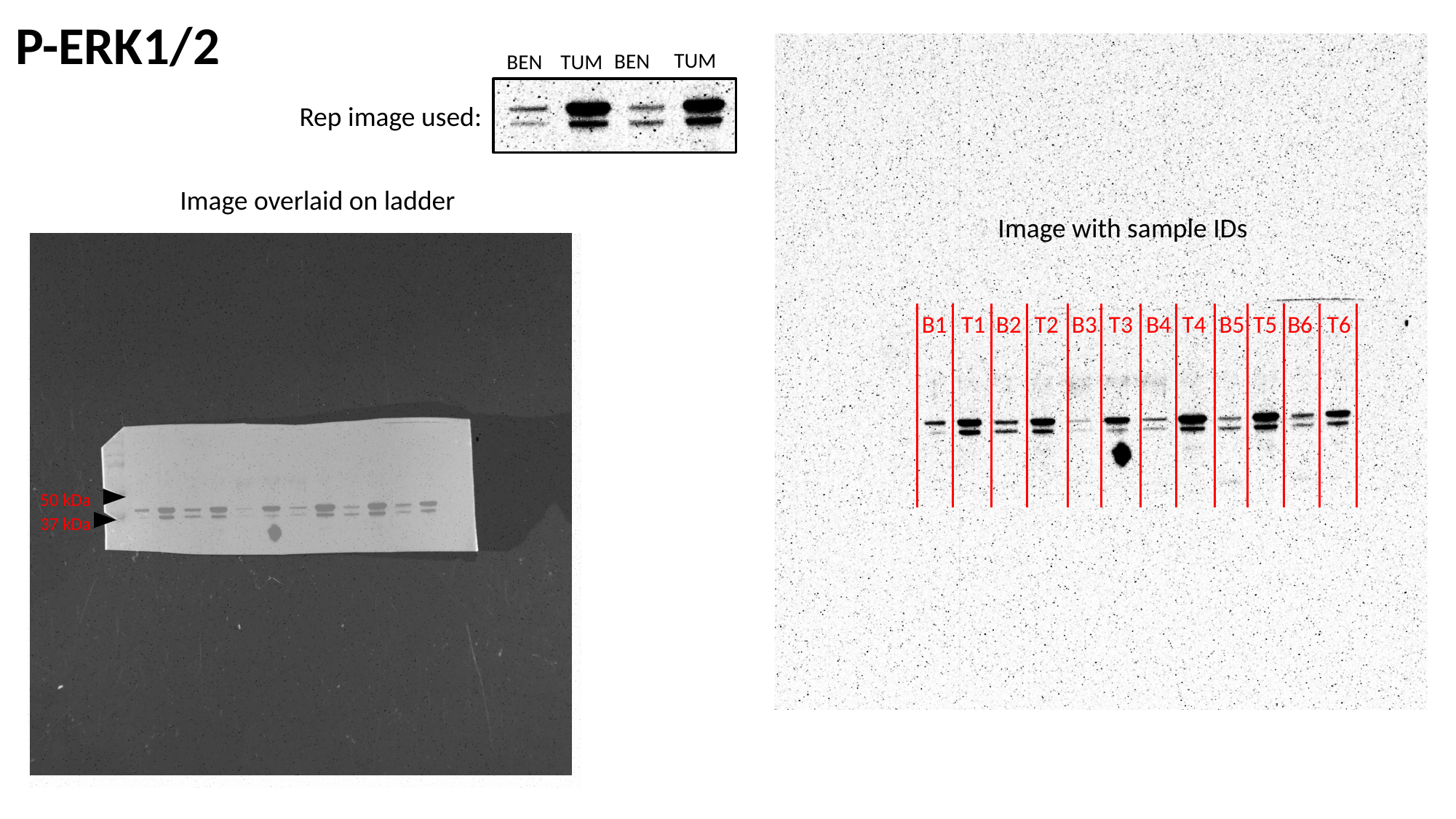

P-ERK1/2
TUM
BEN
BEN
TUM
Rep image used:
Image overlaid on ladder
Image with sample IDs
B1
T1
B2
T2
B3
T3
B4
T4
B5
T5
B6
T6
50 kDa
37 kDa

## Slide 4
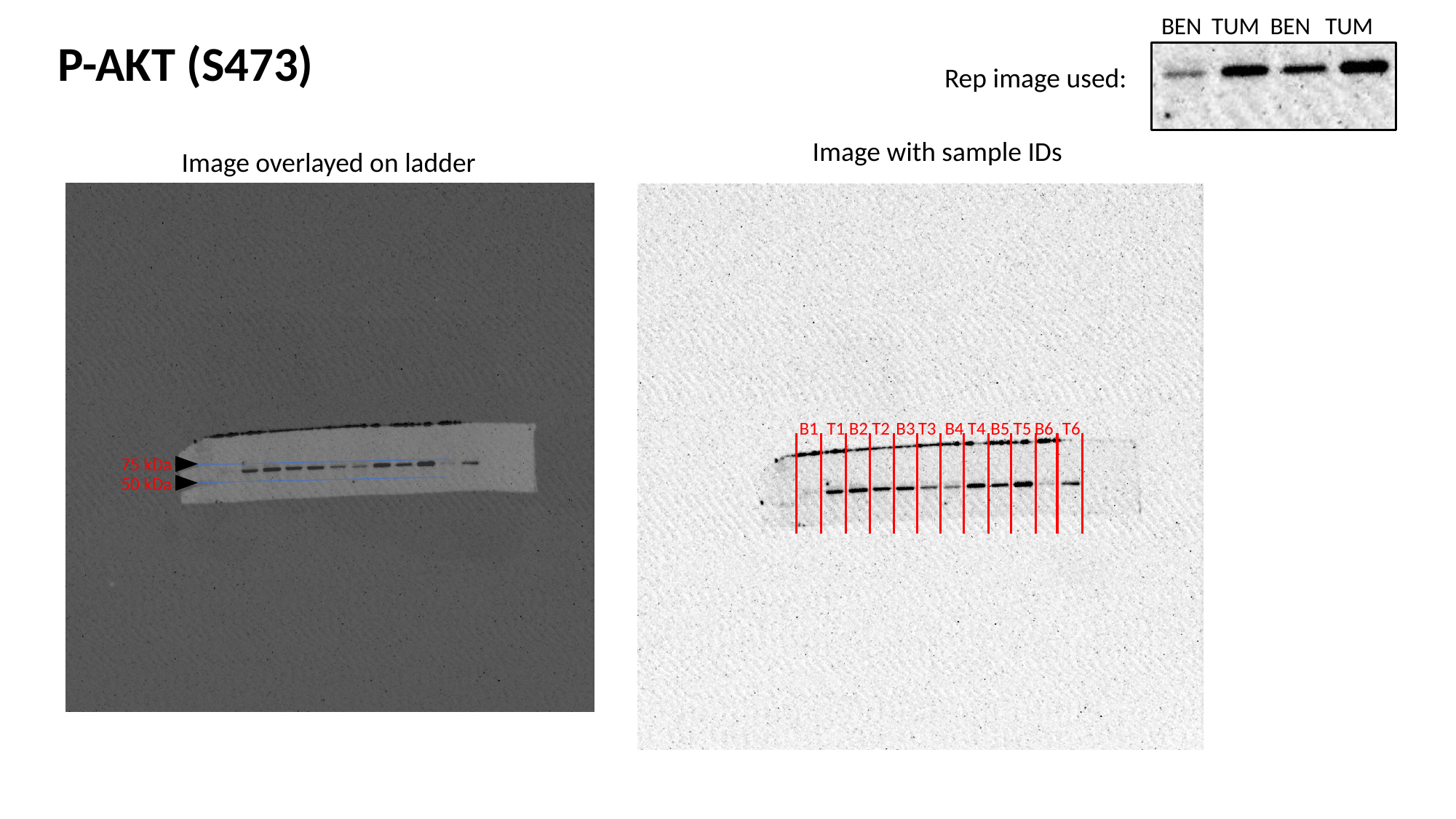

BEN
TUM
BEN
TUM
P-AKT (S473)
Rep image used:
Image with sample IDs
Image overlayed on ladder
B1
T1
B2
T2
B3
T3
B4
T4
B5
T5
B6
T6
75 kDa
50 kDa

## Slide 5
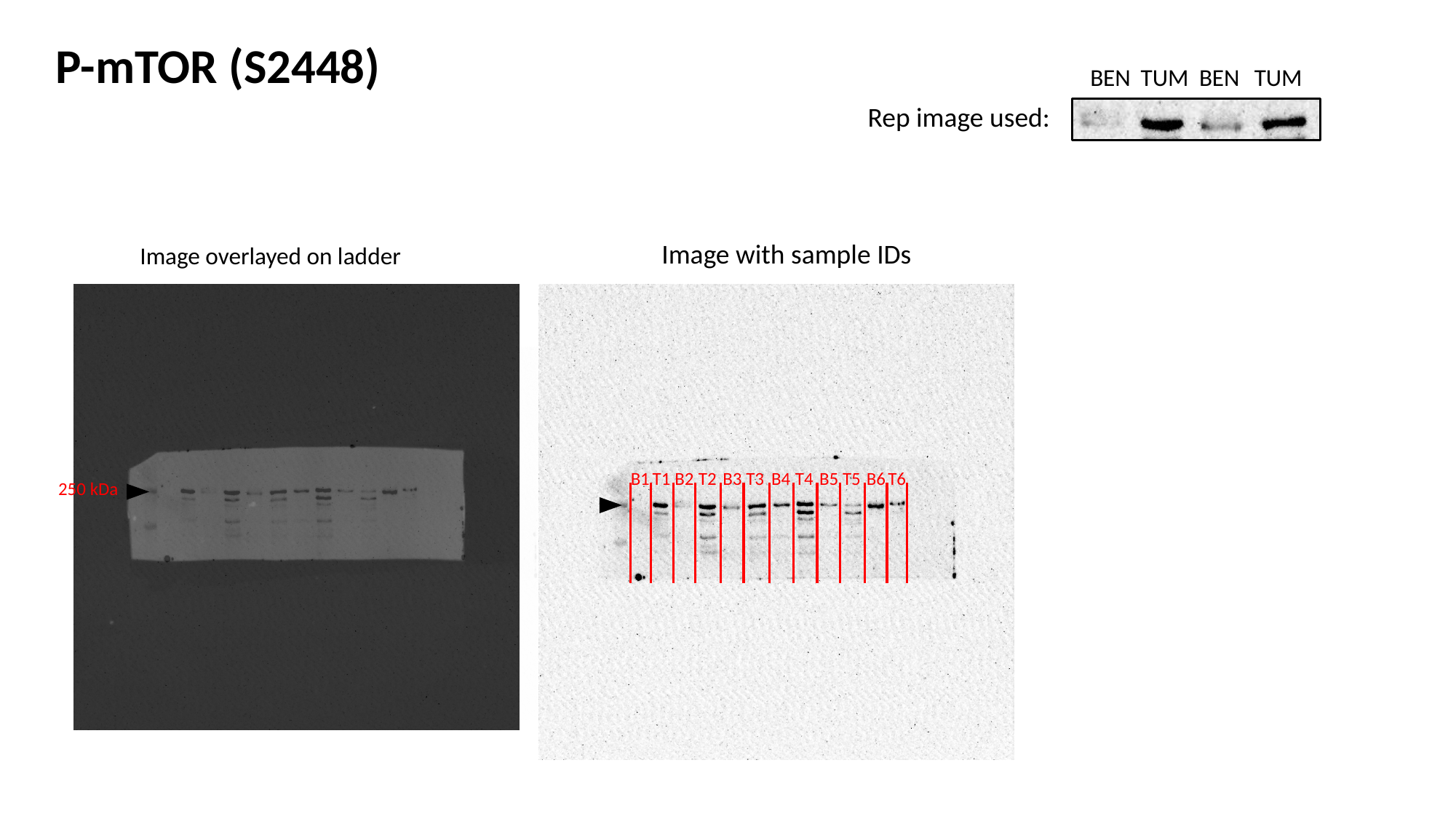

P-mTOR (S2448)
BEN
TUM
BEN
TUM
Rep image used:
Image with sample IDs
Image overlayed on ladder
B1
T1
B2
T2
B3
T3
B4
T4
B5
T5
B6
T6
250 kDa

## Slide 6
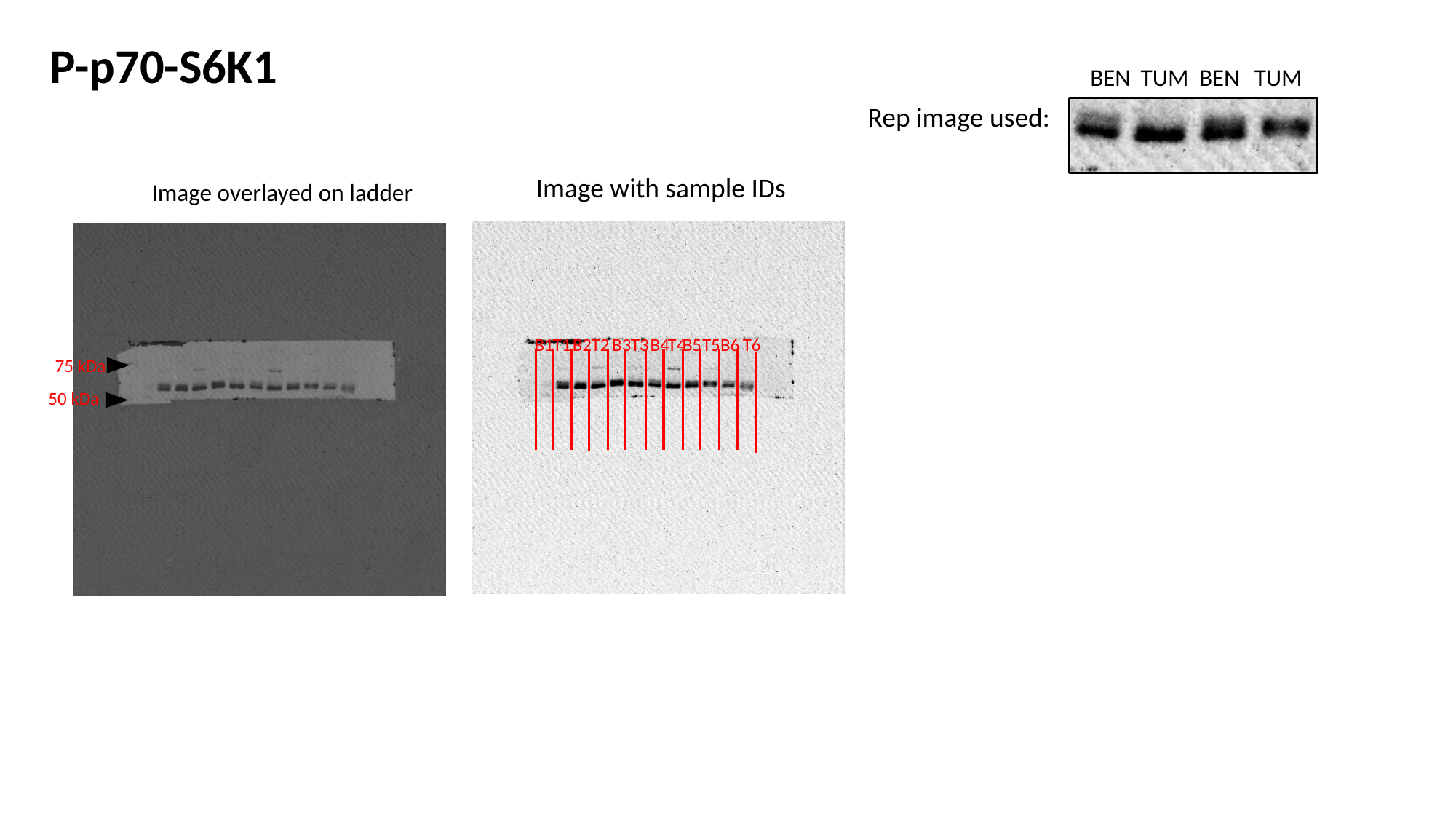

P-p70-S6K1
BEN
TUM
BEN
TUM
Rep image used:
Image with sample IDs
Image overlayed on ladder
B1
T1
B2
T2
B3
T3
B4
T4
B5
T5
B6
T6
75 kDa
50 kDa

## Slide 7
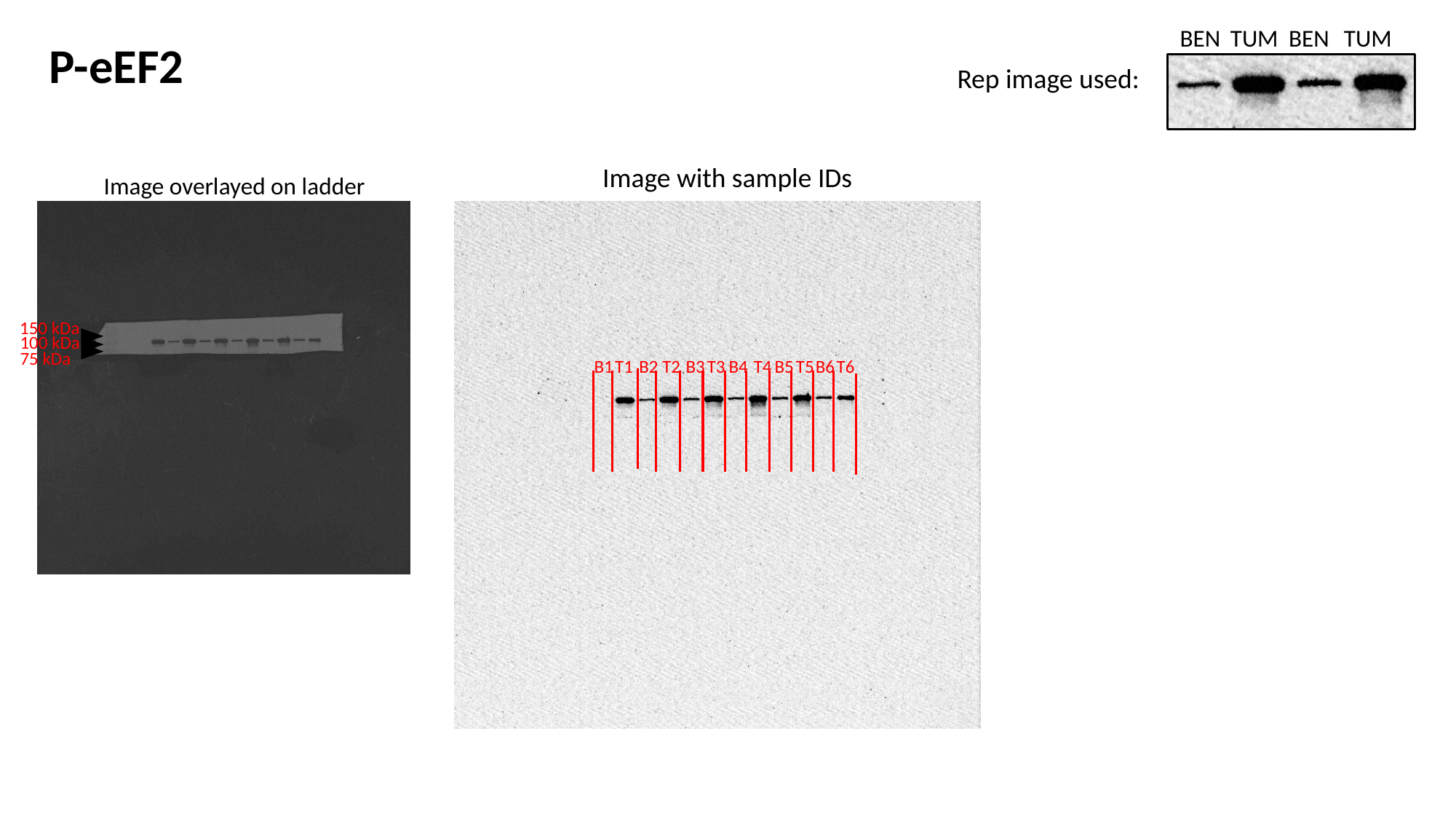

BEN
TUM
BEN
TUM
P-eEF2
Rep image used:
Image with sample IDs
Image overlayed on ladder
150 kDa
100 kDa
75 kDa
B1
T1
B2
T2
B3
T3
B4
T4
B5
T5
B6
T6
