# Supplementary material for: HER2-driven mammary tumorigenesis enhances bioenergetics despite reductions in mitochondrial content
Source: eLife. 2026 May 6;14:RP104079. doi: 10.7554/eLife.104079 (PMC13148823; doi:10.7554/eLife.104079)
Supplement: Figure 5—figure supplement 2—source data 1. [file elife-104079-fig5-figsupp2-data1.zip › Figure 5-figure supplement 2- Source Data 1.pptx]

## Slide 1
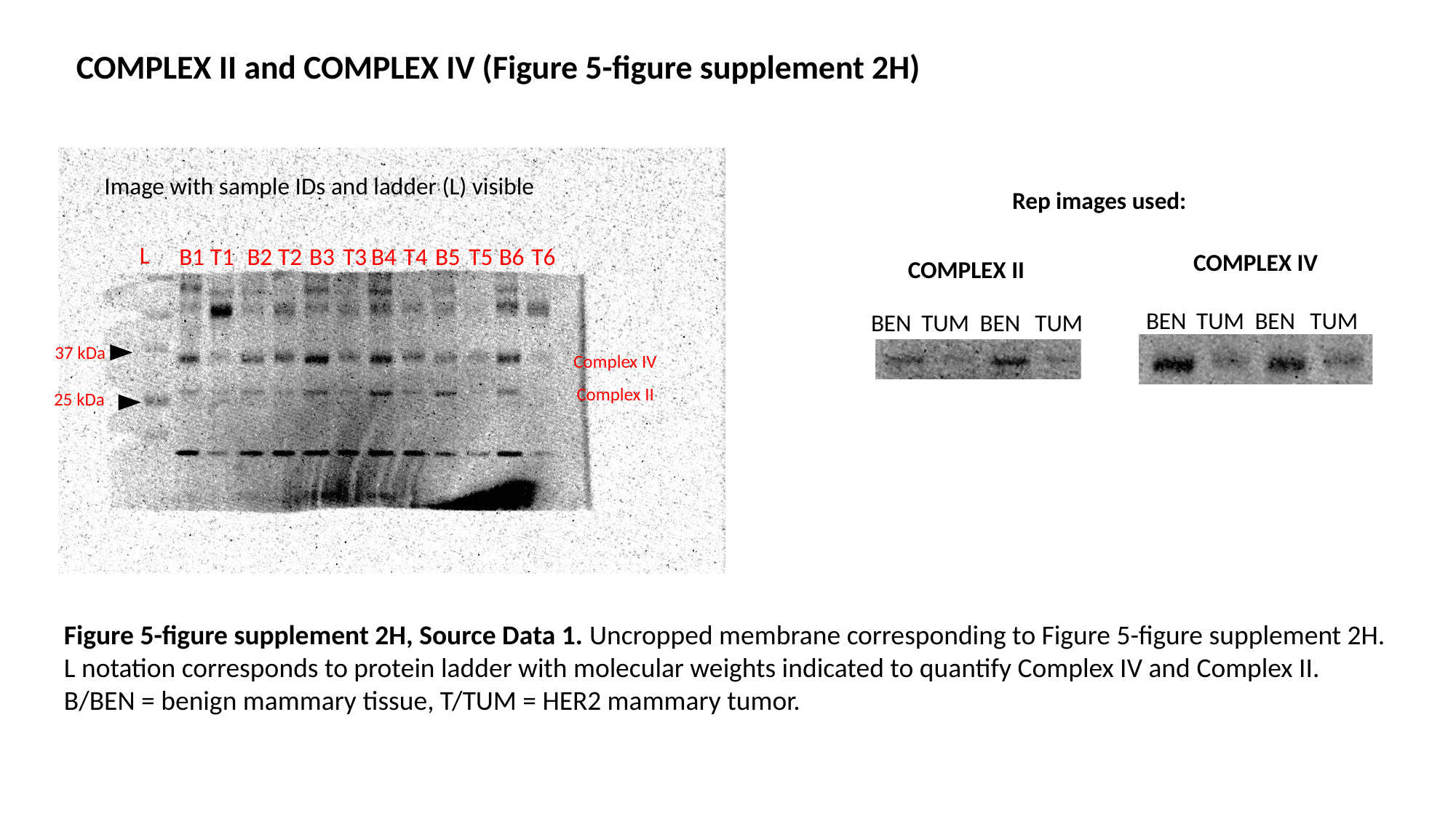

COMPLEX II and COMPLEX IV (Figure 5-figure supplement 2H)
Image with sample IDs and ladder (L) visible
Rep images used:
L
B1
T1
B2
T2
B3
T3
B4
T4
B5
T5
B6
T6
COMPLEX IV
COMPLEX II
BEN
TUM
BEN
TUM
BEN
TUM
BEN
TUM
37 kDa
Complex IV
Complex II
25 kDa
Figure 5-figure supplement 2H, Source Data 1. Uncropped membrane corresponding to Figure 5-figure supplement 2H. L notation corresponds to protein ladder with molecular weights indicated to quantify Complex IV and Complex II. B/BEN = benign mammary tissue, T/TUM = HER2 mammary tumor.
